# Mitigations for extra stimuli of the left ventricular endocardium with the WiSE-CRT System

**DOI:** 10.1016/j.ijcha.2026.101922

**Published:** 2026-04-13

**Authors:** Shunsuke Eguchi, Yoshiyuki Orihara, Ayumi Eguchi, Michael Pfeiffer, John Gorcsan III, John Boehmer, Ryan Wilson

**Affiliations:** Heart and Vascular Institute, Pennsylvania State University College of Medicine, USA

**Keywords:** Resynchronization therapy, Heart failure, Ventricular arrhythmia, Echocardiography, Sonographer, Mechanical index

## Abstract

• The WiSE-CRT System is a novel leadless left ventricular endocardial pacing system.
• Unintentional extra stimuli might occur during TTE after implantation.
• This article describes an alternative imaging protocol to mitigate extra stimuli.

## Graphical abstract

If an apical or near apical electrode position is discovered or extrasystoles occur during echocardiography in patients with the WiSE-CRT System, the ultrasound probe should be removed, and the alternative protocol should be applied.

**Figure:**
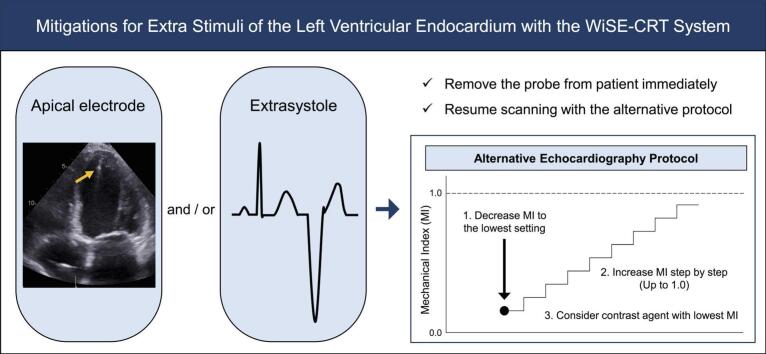

Disclosures

Dr. Gorcsan has received research funding from GE Healthcare, TOMTEC, V-Wave Ltd, EBR Systems, and Canon. These associations had no impact on this study. All other authors have no relationships relevant to the contents of this paper to disclose.

The Wireless Stimulation Endocardially for Cardiac Resynchronization Therapy System (WiSE-CRT System; EBR Systems, Sunnyvale, CA) is a new leadless endocardial system that enables biventricular pacing in patients with heart failure (HF) with reduced ejection fraction who cannot be treated with conventional left ventricular (LV) leads. The safety and efficacy were previously assessed in the Stimulation of the Left Ventricular Endocardium for Cardiac Resynchronization Therapy (SOLVE-CRT trial) [1], [2]. This study revealed that patients who underwent WiSE-CRT placement had improvements in LV function and HF symptoms at 6 months [3]. Approximately 81 % of patients experienced no adverse events related to the device or procedure [4]. Based on the SOLVE-CRT trial, the WiSE-CRT System holds promise as an alternative for conventional CRT [3], [4]. The system received approval from the United States Food and Drug Administration in April 2025, and is expected to be adopted more widely for the treatment of eligible patients.

Transthoracic echocardiography (TTE) is frequently utilized to evaluate patients with HF and generally, ultrasound signal does not elicit extra stimuli [5]. Given the inclusion of an ultrasound sensitive device in the WiSE-CRT System, performing TTE increases the risk of developing extra stimuli. In rare instances, the leadless LV electrode converted ultrasound signal into an electrical signal, generating an extra stimulus. Extra stimuli may result in extrasystoles of asynchronous paced complexes that could potentially develop into more serious ventricular arrhythmias. Extra stimuli refer to unintended electrical stimulation generated when ultrasound energy from the probe is converted into an electrical signal by the electrode. Extrasystole refers to an asynchronous paced complex following such unintended stimulation. Sonographers should have awareness of the potential for extra stimuli and how to mitigate it before performing TTE on patients with the WiSE-CRT System.

Conventional CRT devices directly transmit electronic signals to the LV via the coronary sinus lead. In contrast, the WiSE-CRT System utilizes ultrasound energy to deliver a signal to a leadless electrode and provide LV stimulation synchronized to right ventricular pacing. The WiSE-CRT System is composed of (1) an ultrasound transmitter, (2) a battery connected to the transmitter, and (3) an electrode. This technology utilizes ultrasound energy to power its electrode, enabling its leadless design for LV implantation.

During TTE in patients with the WiSE-CRT System, the risk of extra stimuli is increased. Overall, in the 459 patients worldwide who have been implanted with the WiSE-CRT System, 13 extra stimuli events during TTE have occurred, 2.8 % of patients. Two of these events have been previously described in published reports [3], [6]. Extrasystoles occurred during TTE and resolved promptly after removal of the ultrasound probe from the chest wall without sustained ventricular arrhythmia.

There have been no reports of extra stimuli with either transesophageal or intracardiac echocardiography despite the mandated use of these modalities during the implant procedure. This is most likely because the WiSE-CRT System operates at 0.921 MHz while transesophageal and intracardiac echocardiography are significantly higher and above the frequency of maximum sensitivity for the system.

Several factors have been identified that can increase the potential risk for extra stimuli. These include (1) apical or near apical electrode position (Fig. 1), (2) short time interval after electrode implant, (3) imaging from apical views (resulting in the ultrasound probe being in very close proximity to the electrode), (4) high power settings (displayed as mechanical index [MI]), (5) low frequency ultrasound.

**Fig. 1:**
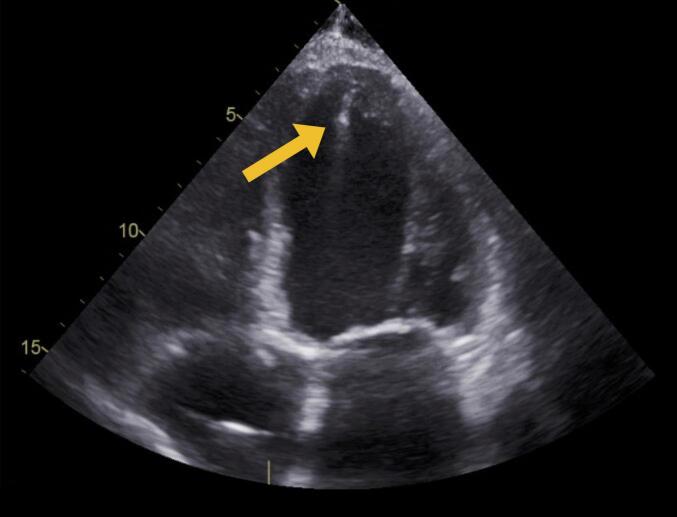
Representative case with apical electrode placement. TTE image obtained from the apical window, demonstrating an apical electrode placement (indicated by arrow). TTE, transthoracic echocardiography.

Patients with the WiSE-CRT System should be monitored with electrocardiography during TTE. If an apical or near apical electrode position is discovered or extrasystoles occur, it is recommended to follow the labeling, removing the probe from the patient immediately, and resuming scanning using the following protocol (Fig. 2).

**Fig. 2:**
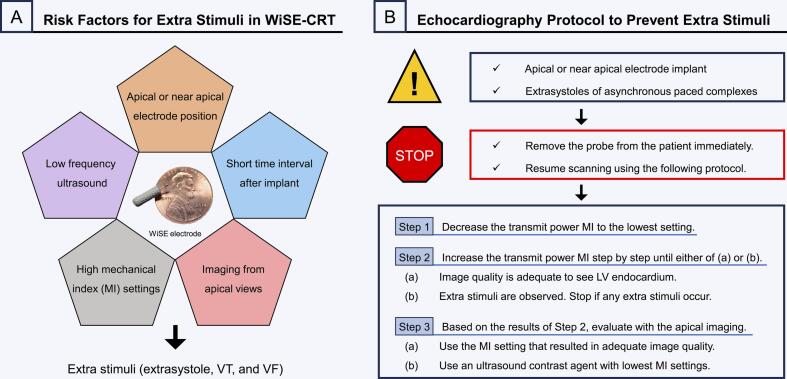
Risk factors for extra stimuli in the WiSE-CRT System and alternative echocardiographic imaging protocol to prevent extra stimuli. (A) Major risk factors for extra stimuli. (B) The protocol should be applied if an apical or near apical electrode position is discovered or extrasystoles are detected during TTE in patients with the WiSE-CRT System. TTE, transthoracic echocardiography.

Step 1. Decrease the transmit power MI to the lowest setting. Reduce the MI to the lowest possible setting as done with echocardiographic contrast imaging in routine clinical practice, usually an MI much less than 1.0.

Step 2. Increase the transmit power MI step by step until either of (a) or (b) occurs. (a) Image quality is adequate to see LV endocardium for LV volume determination. (b) Extra stimuli are observed. Stop if any extra stimuli occur. In general, it is not advised to increase MI over 1.0.

Step 3. Based on the results of Step 2, evaluate with the apical imaging. (a) If no extra stimuli occur in Step 2, use the MI setting for routine imaging that resulted in adequate image quality. (b) If extra stimuli occur in Step 2, use an ultrasound contrast agent with the lowest MI setting to allow evaluation of LV cavity size and function.

In this protocol, lowering the MI is a central technique utilized to decrease the risk of extra stimuli. MI ranges are defined as follows: very low, values < 0.2; low, values < 0.3; intermediate, values of 0.3 to 0.5, and high is any MI value that exceeds 0.5 [7]. Contrast echocardiography, which is recommended in Step 3, utilizes commercially available ultrasound contrast agents to improve LV opacification. These ultrasound contrast agents require very low MI setting to optimize visualization and are therefore favorable to optimize image quality and mitigate risk of developing extra stimuli particularly during TTE in patients with the WiSE-CRT System.

In summary, conventional TTE imaging may create the potential for ventricular extra stimulations in a small number of susceptible patients. To address this concern, our proposed approach to TTE imaging was developed based on device functionality, knowledge of ultrasound physics, and expert consensus. Only a small number of events have been reported, and it is beyond the scope of these data to demonstrate reduced risk. This article aims to increase recognition of potential extra stimuli and provide practical recommendations to mitigate this risk. These recommended techniques have been endorsed by the manufacturer and the echocardiographic core laboratory (Pennsylvania State University College of Medicine, Hershey, PA, USA). Following the labeling instructions and understanding appropriate imaging techniques are important for recognizing and mitigating the potential for extra stimuli. This article provides an echocardiographic imaging protocol to allow safe assessment of cardiac function in patients that are susceptible to extra stimuli.

## Declaration of competing interest

The authors declare that they have no known competing financial interests or personal relationships that could have appeared to influence the work reported in this paper.
